# Reliability and diagnostic accuracy of corrected slack angle derived from 2D-SWE in quantitating muscle spasticity of stroke patients

**DOI:** 10.1186/s12984-022-00995-8

**Published:** 2022-02-05

**Authors:** Junyan Cao, Yang Xiao, Weihong Qiu, Yanling Zhang, Zulin Dou, Jie Ren, Rongqin Zheng, Hairong Zheng, Zhaocong Chen

**Affiliations:** 1 Department of Medical Ultrasonics, The Third Affiliated Hospital of Sun Yat-Sen University, Sun Yat-Sen University, 600 Tianhe Road, Guangzhou, 510630 China; 2 Institute of Biomedical and Health Engineering, Shenzhen Institutes of Advanced Technology, Chinese Academy of Sciences, 1068 Xueyuan Avenue, Shenzhen, 518055 China; 3 Department of Rehabilitation Medicine, The Third Affiliated Hospital of Sun Yat-Sen University, Sun Yat-Sen University, 600 Tianhe Road, Guangzhou, 510630 China; 4 Paul C. Lauterbur Research Center for Biomedical Imaging, Institute of Biomedical and Health Engineering, Shenzhen Institutes of Advanced Technology, Chinese Academy of Sciences, 1068 Xueyuan Avenue, Shenzhen, 518055 China

**Keywords:** Corrected slack angle, Stroke, Muscle spasticity, Medial gastrocnemius, Two-dimensional shear wave elastography

## Abstract

**Background:**

To explore the feasibility of corrected slack angle acquired from two-dimensional shear wave elastography (2D-SWE) for quantitating the spasticity of medial gastrocnemius (MG) in stroke patients.

**Methods:**

Consecutive stroke patients with spastic MG and matched healthy controls were recruited. Intra- and interobserver reliability of 2D-SWE measurement were evaluated, and the correlation between corrected slack angle and modified Ashworth scale (MAS) score was examined. The corrected slack angle before and after botulinum toxin A (BoNT-A) injection was compared and its diagnostic performance in classifying the severity of spasticity were assessed with receiver operating characteristic (ROC) curve analysis.

**Results:**

The intra- (0.791 95% CI 0.432–0.932) and interobserver (0.751 95% CI 0.382–0.916) reliability of slack angle acquired with 2D-SWE were good. Significant correlation was found between corrected slack angle and MAS score (R = − 0.849, *p* < 0.001). The corrected slack angle increased after BoNT-A injection. The cutoff value of MAS ≥ 3 had the highest sensitivity (100%) and specificity (93.33%). The positive predictive value (PPV) for classification of MAS ≥ 1+ and the negative predictive value (NPV) for classification of MAS ≥ 3 were greater than 90%.

**Conclusion:**

2D-SWE was a reliable method to quantitate the post-stroke spasticity. The corrected slack angle had advantage in classifying the severity of spasticity, especially in early identification of mild spasticity and confirmation of severe spasticity.

**Supplementary Information:**

The online version contains supplementary material available at 10.1186/s12984-022-00995-8.

## Background

Spasticity is a common complication of central nervous system injury including cerebrovascular accident, brain trauma and spinal cord injury, etc. [1]. It has been estimated that spasticity affects approximately 19% to 45% of patients suffering from stroke [2, 3]. Quantitation of spasticity is necessary, in order to assess the impact of therapies and to determine appropriate medicine dosage. The modified Ashworth scale (MAS) is the most feasible measurement of spasticity in clinical practice [4]. However, its dependency on subjective assessment restricts its application. Magnetic resonance elastography is capable of objectively evaluating the spasticity of individual muscle [5], but it is limited by a variety of contraindications (e.g., cardiac pacemaker, metal implants, claustrophobia).

Ultrasound elastography had been widely used to examine tissue elasticity in various organs, including breast [6], prostate [7] and liver [8]. Recently, it has been developed as a quantitative method for the evaluation of skeletal muscle. Several studies have attempted to investigate the mechanical properties of the spastic muscles in post-stroke patients [9–11]. However, various measurements (strain ratio [9], elasticity index [10], shear elastic modulus [11]) were reported because of different elastography techniques they used. As the newest modality of ultrasound elastography that uses acoustic radiation force and generate quantitative elastograms [12], although the technical assumption may not perfectly be met in skeletal muscle because skeletal muscle is not a homogeneous or isotropic material [13], two-dimensional shear wave elastography (2D-SWE) has also been used in evaluation of skeletal muscle cautiously. But its standard protocol is still lacking, which makes it difficult to achieve consensus on the cut-off elastic value of spastic muscles. Besides, the correlation between shear modulus and the clinical assessment such as MAS for stroke patients remains equivocal [14, 15].

Slack angle, defined as the angle of joint from where the muscle becomes tensioned and the shear modulus begins to rise as the joint was passively moved, has been a crucial parameter to characterize the mechanical property of skeletal muscles [16, 17]. Because skeletal muscle is a kind of active and deformable tissue, continuous 2D-SWE recording would make sense. It has been revealed that the slack angle of gastrocnemius occurred at a more plantarflexed angle in stroke patients than control subjects [18]. However, the slack angle was visually determined in previous studies [17–19]. Its inter- and intraobserver reliability and its correlation with clinical assessment have not been reported yet. Moreover, the inter-individual variability of muscle elasticity, caused by gender, age, physical activity and biological structure [20], may be a confounding factor when using the slack angle to evaluate the spasciticy of muscle. Instead of comparing with the spastic slack angle directly, we suggest that it would be reasonable to propose the corrected slack angle, which was performed a self-correction by subtracting the shear modulus of the unaffected muscle from that of the spastic muscle. It could be beneficial to rule out the confounding factors related to the passive extensibility of skeletal muscle.

Therefore, the present study aims to explore the feasibility of quantitating the spasticity of stroke patients with corrected slack angle acquired from 2D-SWE. For this purpose, whether corrected slack angle correlated with MAS and its change after botulinum toxin A (BoNT-A) injection are examined, as well as its diagnostic performance for classifying the severity of spasticity.

## Methods

### Subjects

**Fig. 1:**
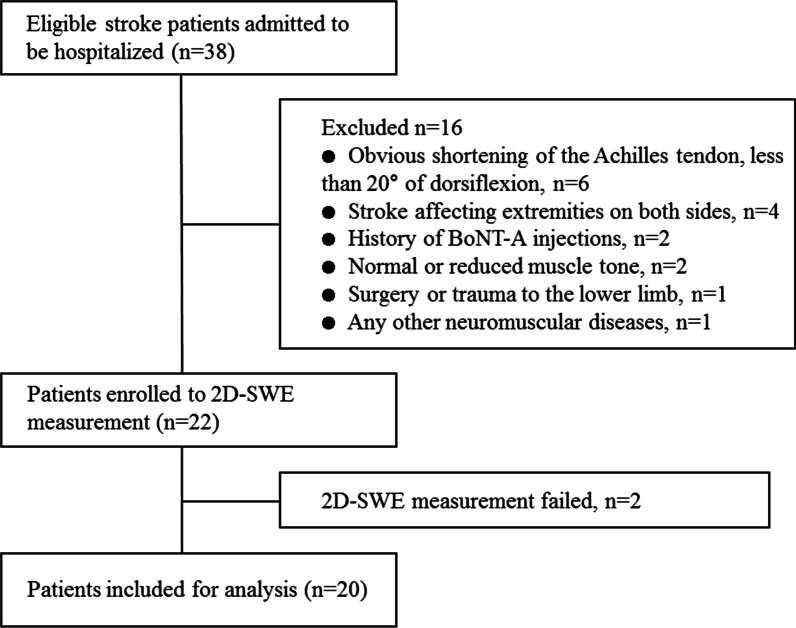
Flowchart of stroke patients included in the study

Consecutive patients admitted to the Department of Rehabilitation Medicine in The Third Affiliated Hospital of Sun Yat-sen University from May 2019 to February 2020 with a confirmed diagnosis of stroke by neuroimaging within 6 months were enrolled. Exclusion criteria were listed in Fig. 1. Patients classified as MAS 4 were not recruited because the ankle of those patients was rigid and could not be moved. The passive range of motion (ROM) of included patients were listed in Additional file 1: Tables S1 and S2. Age- (≤ 2-year difference), gender and footedness-matched healthy controls were recruited from the community. The footedness was determined by (1) the preferred foot used to kick the ball on the floor and (2) the leg selected to put most of body weight on during relaxed standing. None of them reported a history of trauma or surgery on lower extremity, neuromuscular disorders and medication that may have affected muscle movement. Written informed consent was obtained from all participants, and the study protocol was approved by institutional review board at The Third Affiliated Hospital of Sun Yat-sen University in Guangzhou.

### Clinical assessment

MAS was assessed separately by two physical therapists with over 10 years of professional experience blinded to the 2D-SWE values. The MAS score would be achieved based on an agreement by these two therapists. The MAS was rated as follows: 0 = no increased resistance; 1 = minimal resistance at the end of range of motion (ROM); 1+ = minimal resistance throughout less than half of the ROM; 2 = clear resistance throughout most of the ROM; 3 = strong resistance, passive movement is difficult; 4 = rigid [21]. Participants were required to rest for 5 min before assessment.

### 2D-SWE measurement

**Fig. 2:**
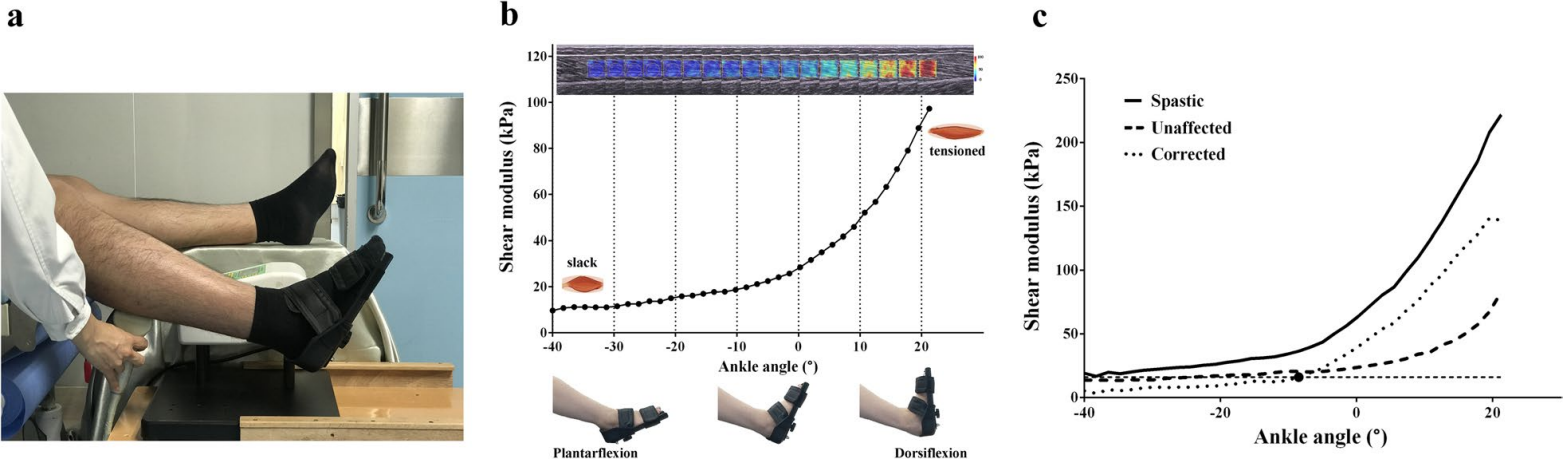
**a** Experimental setup overview. Participants lied in supine position comfortably with the knee flexed at 30°. The ankle was passively moved by a continuous passive motion device from 40° plantarflexion to 21.5° dorsiflexion. The probe of an ultrasonic scanner was placed on the thickest part of MG with minimal pressure. Surface electromyogram was monitored in real time (electrodes were attached to the medial gastrocnemius and could not be seen). **b** A typical shear modulus-angle relationship curve of MG acquired from single measurement trial of 2D-SWE. **c** The procedure of self-correction and locating the corrected slack angle. Solid line and dash line denote the modulus-angle curve of spastic MG and unaffected MG respectively. The dotted line denotes the corrected modulus-angle curve obtained by subtracting the dash line from the solid line. The solid dot is the intersection of the threshold line calculate by 3-SD criterion and the corrected curve, denoting the location of the corrected slack angle

2D-SWE measurement was performed within 2 days after MAS assessments at the same time period (between 3:00 and 5:00 pm). The participant lied in supine position comfortably with the knee flexed at 30° because the supine position was more tolerable than prone position for the patients and they were able to remain relaxed under this position (Fig. 2a). The isokinetic ankle movement from 40° plantarflexion (PF) to 21.5° dorsiflexion (DF) was conducted by a continuous passive motion (CPM) device (Y&Z medical Inc.) running with the angular velocity of 1.75°/s, which was one of the default velocity of the CPM. This relative slow velocity might made our study comparable with other studies where the angular velocity was set as 1°/s [16] or 2°/s [18, 19, 22].

An ultrasonic scanner (Aixplorer; Supersonic Imagine) equipped with a linear array transducer (4–15 MHz, SuperLinear 15–4) was used. The probe was placed on the thickest level of the medial gastrocnemius (MG) with minimal pressure and parallel to the longitudinal axis of the muscle. The supersonic shear imaging mode (musculo-skeletal preset) was launched and a 10 mm × 10 mm color-coded sample box was placed in the center of MG belly without inclusion of vessels or fascial borders. A cine loop of 35-s was captured to record the continuous change of elastogram along with the ankle movement. Surface electromyogram (ME6000; Mega Electronics) was monitored in real time to ensure that the MG was free of contraction at the beginning stage. Both legs of the stroke patients were measured. For the healthy controls, the same side corresponding to the spastic side of the patient they matched was chosen. Each SWE session consisted of three trials separated by 1 min interval. Before each session, there was a 5-min-break when participants were instructed to remain as calm and relax as possible.

To test the intra- and interobserver reliability, 9 controls and 10 patients were selected using complete randomization method. Considering the patient's limited tolerance to repeated examination, five of them was re-examined by another examiner (Y. L. Zhang) within 24 h after the initial measurement, and the others was re-examined by the first examiner (J. Y. Cao) within 3 days. Both examiners had extensive experience (> 3 years) with 2D-SWE and blinded to the MAS scores. The 9 controls were re-examined twice by both examiner and not allowed to perform strength training between sessions. For these participants receiving reliability test, each SWE session consisted of two trials.

### BoNT-A injection

Five patients received BoNT-A injection at the spastic gastrocnemius. The dosage targeting the MG was adapted to the patient’s individual characteristic, ranging from 80 to 120 units. The injection was performed by a physician (W. H. Qiu) with more than 10 years of experience. SWE measurement and MAS assessment were repeated 2 weeks after injection.

### Data analysis

The cine loops were saved as ‘avi’ format and processed in Aixplorer scanner software. A circular region of interest (ROI) with a 10 mm diameter was drawn inside the color-coded sample box for the first frame of every second. In a few cases, the ROI was adjusted to avoid the blank in the sample box. The mean value of the shear modulus (in kPa) was calculated within the ROI and averaged across trials. Because the ankle was moved at a constant velocity, each second could be converted to a corresponding angle. Therefore, a modulus-angle relationship curve could be drawn for each session (Fig. 2b). To describe the modulus-angle curve via the mean values of shear modulus from the initial, middle and final stage of the curve, three sections of the curve centered on PF 25° (− 29.5° to − 20.75°), PF 5° (− 10.25° to − 1.5°) and DF 15° (10.75° to 19.5°) (minus value represented plantarflexion), separated by 10° approximately, were extracted and their averaged modulus was acquired for multiple comparisons and reliability tests.

Instead of visually inspection, we proposed a three standard deviation (3-SD) criterion [23] to determine the slack angle objectively. The slack angle was located as where an ascending of modulus that exceeded a value of three standard deviations of its previous mean modulus and such tendency sustained for the following two angles. Because the color box of shear modulus were obtained at 1 Hz, it could lead to a 1-s delay between the measured shear modulus and the ankle angle. To offset this delay, we proposed the formula of 3-SD criterion as:

$$x_{n + 1} > \left( {\overline{x}_{n} + 3\upsigma _{n} } \right),$$

where n denotes the order corresponding to the ankle angle, $x_{n}$ denotes the shear modulus acquired at that ankle angle, the standard deviation of shear modulus from the first value to the nth value $\sigma_{n} = \sqrt {\frac{1}{n - 1}\mathop \sum \limits_{i = 1}^{n} \left( {x_{i} - \overline{x}_{n} } \right)^{2} }$ and the mean of shear modulus from the first value to the nth value $\overline{x}_{n} = \frac{{\mathop \sum \nolimits_{i = 1}^{n} x_{i} }}{n}$. Because $x_{n + 1}$ was actually the shear modulus matched with n, hence when $x_{n + 1}$ was larger than $\overline{x}_{n} + 3\sigma_{n}$ and the relationship of this inequality also existed for the next two orders, the angle corresponding to n was defined as the slack angle. It was also available to be located on the corrected modulus-angle curve, which was derived from the modulus of the spastic muscle minus that of the unaffected muscle (Fig. 2c).

### Statistical analysis

Demographic and clinical characteristics of the participants were reported using descriptive statistics (mean, standard deviation, and percentage). The Mann–Whitney test or independent samples t-test was used to compare the means of two groups according to the assumptions of normality tested with Shapiro–Wilk’s test. A two-way repeated analysis of variance (RM-ANOVA) was performed to determine position (PF 25°, PF 5° and DF 15°) and group (patients and controls) effects on shear modulus. The Spearman correlation coefficient was applied to test the correlation between MAS score and shear modulus or slack angle. Intra- and interobserver reliability were examined with the two-way random intraclass correlation coefficient (ICC), which was classified as poor (0.00–0.50), moderate (0.50–0.75), good (0.75–0.90), and excellent (> 0.90). The diagnostic performance of slack angle for classifying the severity of spasticity was assessed with receiver operating characteristic (ROC) curves. The areas under the ROC curves (AUROCs) were compared using the Delong test [24]. Optimal cutoff values were established by maximizing Youden’s index on the ROC. The sensitivity, specificity, positive predictive value (PPV), negative predictive value (NPV), positive likelihood ratio (LR+), and negative likelihood ratio (LR−) based on the optimal cutoff values were calculated. P values < 0.05 were considered statistically significant. Statistical analyses were carried out using SPSS software (Version 13.0) and MedCalc Statistical Software (version 12.7).

## Results

During the recruitment, 38 eligible stroke patients were enrolled, of which 16 patients were ruled out as shown in Fig. 1. Two patients had difficulty to maintain the position and failed to complete the SWE measurement. Eventually, 20 patients and 20 healthy controls were included for analysis. The demographic and clinical characteristics of the participants were shown in Table 1. There was no statistical difference in age and BMI between two groups (*p* ≥ 0.596).

**Table 1:** Characteristics of the study participants

Characteristics	Patient group (*n* = 20)	Control group (*n* = 20)	*p* value
Gender			1.000
Male	13 (65%)	13 (65%)	
Female	7 (35%)	7 (35%)	
Age (years)	52.30 ± 11.55	53.25 ± 11.54	0.980
BMI (kg/m^2^)	23.04 ± 1.76	22.11 ± 1.96	0.596
Days since stroke	79.15 ± 35.37	*NA*	
Stroke etiology			
Ischemic	12 (60.00%)	*NA*	
Hemorrhage	8 (40.00%)	*NA*	
Affected side			
Right	9 (45%)	*NA*	
Left	11 (55%)	*NA*	
MAS			
1	4 (20%)	*NA*	
1+	6 (30%)	*NA*	
2	5 (25%)	*NA*	
3	5 (25%)	*NA*	

Data are means ± standard deviation or numbers of participants and data in parentheses are percentages

*BMI* body mass index, *MAS* modified Ashworth scale, *NA* not applicable

### Reliability analysis

The intraobserver reliability of the shear modulus was moderate in the PF 5° (0.739), good in the PF 25° (0.802) and excellent in the DF 15° (0.984) (Table 2), while the interobserver reliability was excellent under all ankle position (0.922–0.994) (Table 3). The slack angle was unavailable to calculate for one healthy control, therefore the reliability of slack angle was analyzed for 13 participants. As presented in Tables 2 and 3, both intra- (0.791) and interobserver (0.751) reliability of slack angle were good.

**Table 2:** Intraobserver reliability analysis of slack angle and shear modulus in three different ankle positions

Measurement	1st Examination	2nd Examination	ICC (95% CI)
Shear modulus (kPa) (n = 14)			
PF 25°	12.25 ± 1.74	11.80 ± 1.73	0.802 (0.496–0.932)
PF 5°	21.23 ± 4.35	20.79 ± 3.76	0.739 (0.363–0.908)
DF 15°	64.87 ± 20.88	63.25 ± 18.69	0.984 (0.946–0.995)
Slack angle (°) (n = 13)	− 2.98 ± 8.23	− 0.15 ± 9.24	0.791 (0.432–0.932)

Data are means ± standard deviation

*ICC* intraclass correlation coefficient, 95% confidence interval; *PF* plantarflexion, *DF* dorsiflexion

**Table 3:** Interobserver reliability analysis of slack angle and shear modulus in three different ankle positions

Measurement	Examiner 1	Examiner 2	ICC (95% CI)
Shear modulus (kPa) (n = 14)			
PF 25°	13.36 ± 3.32	13.04 ± 3.27	0.922 (0.783–0.974)
PF 5°	23.07 ± 7.58	23.21 ± 8.06	0.961 (0.884–0.987)
DF 15°	71.63 ± 39.23	72.20 ± 37.54	0.994 (0.983–0.998)
Slack angle (°) (n = 13)	− 0.19 ± 10.47	− 2.31 ± 10.73	0.751 (0.382–0.916)

Data are means ± standard deviation

*ICC* intraclass correlation coefficient, 95% confidence interval, *PF* plantarflexion, *DF* dorsiflexion

### Comparison between patients and controls

**Fig. 3:**
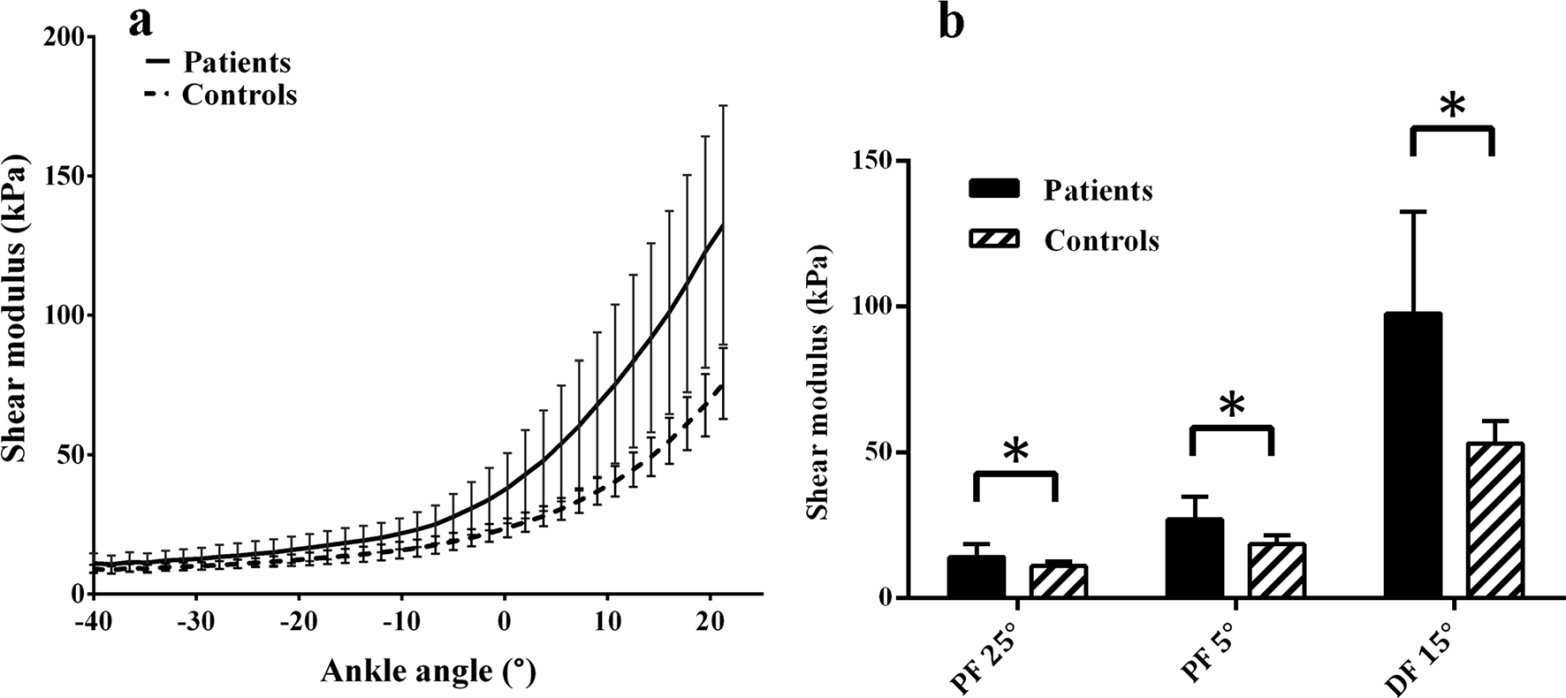
**a** Averaged modulus-angle curves of stroke patients and healthy controls. The error bars indicate the standard deviation from the average modulus for each ankle angle. **b** T-Bar plots showing the difference of shear modulus between stroke patients and healthy controls at the ankle positions of PF 25°, PF 5° and DF 15°. Error Bars indicate standard deviation and whiskers indicate significant difference (*p* < 0.05)

**Fig. 4:**
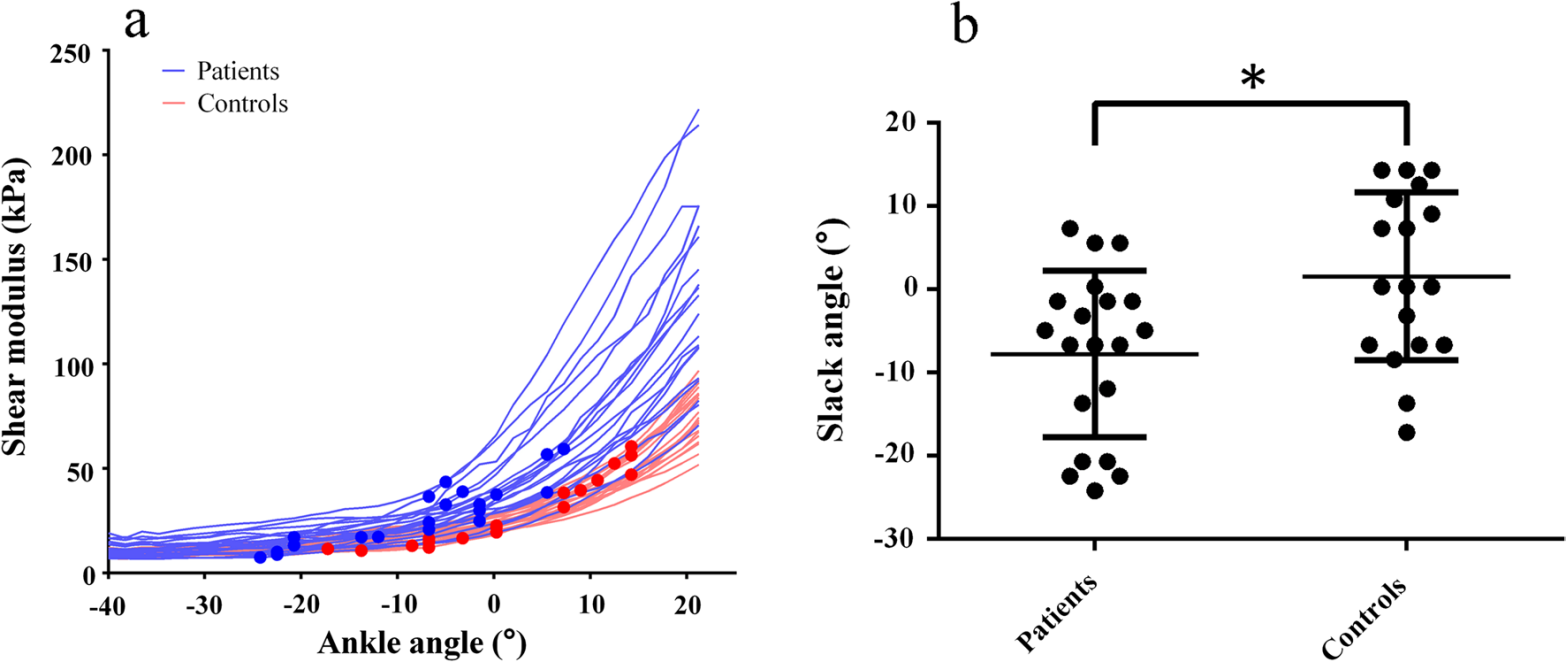
**a** Individual modulus-angle curves of MG in stroke patients and healthy controls. The solid dots denote the locations of slack angle. **b** Scatter plots of slack angle in stroke patients and healthy controls. The horizontal bar represents mean value and error bar represents standard deviation

A two-way RM-ANOVA was used to compare the shear modulus of three ankle positions between patients and controls. The assumptions of normality assessed with the Shapiro–Wilks test were met for all subsets of the data (*p* ≥ 0.164). Significant main effects of position (F(2,76) = 275.917, *p* < 0.001) and group (F(1,38) = 29.715, *p* < 0.001) were revealed. However, because significant interaction was also found between position and group (F(2,76) = 31.022, *p* < 0.001), separate comparisons by ankle position between two groups were performed before post-hoc tests. It showed that the shear modulus of patients' MG was significantly larger than that of healthy controls in all three positions (*p* ≤ 0.006) (Fig. 3; Additional file 1: Table S3). In terms of the slack angle, it showed up significant earlier (i.e. more plantarflexed) in stroke patients (− 7.8 ± 9.99°) compared to controls (1.51 ± 10.09°) (*p* = 0.007) (Fig. 4).

### Correlation with MAS score

**Fig. 5:**
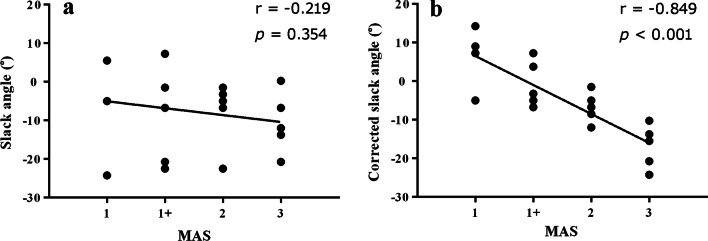
**a** Correlation between slack angle and MAS score in stroke patients. Two points were overlapped at 5° for MAS 1 and at − 1.5° for MAS 1+. **b** Correlation between corrected slack angle and MAS scores in stroke patients. Two points were overlapped at 5° for MAS 1+

**Fig. 6:**
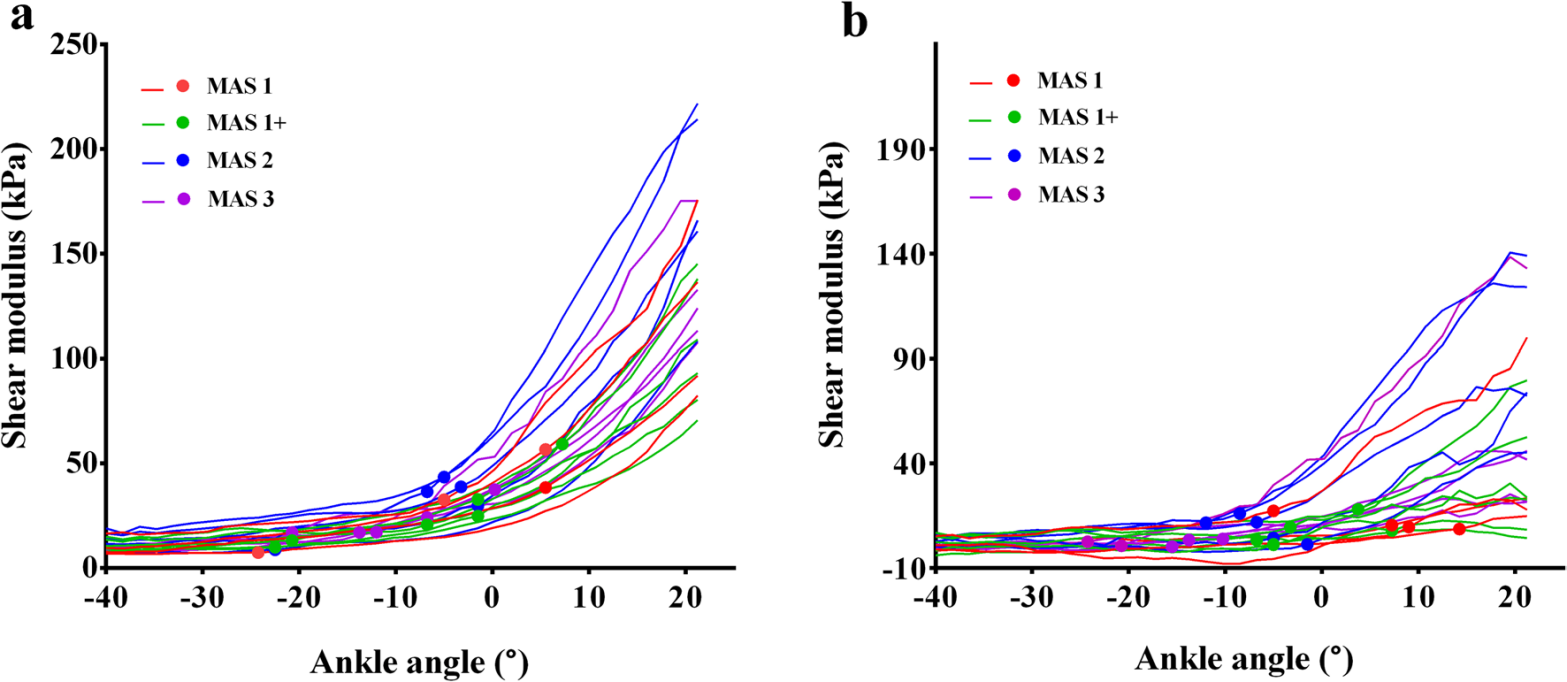
**a** Individual modulus-angle curves of spastic MG in stroke patients of whom the MAS score indicated by different colors. The solid dots denote the locations of slack angle. **b** Individual corrected modulus-angle curves of stroke patients of whom the MAS score indicated by different colors. The solid dots denote the locations of corrected slack angle

Although the shear modulus and the slack angle of stroke patients were different from those of healthy controls, neither shear modulus nor slack angle significantly correlated with MAS score (Figs. 5a, 6a and Additional file 1: Fig. S1, Table S4). However, with self-correction to the shear modulus of the spastic MG, there was significant correlation between corrected slack angle and MAS score (R = − 0.849, *p* < 0.001) (Figs. 5b and 6b; Additional file 1: Table S4).

### Changes after BoNT-A injection

The Additional file 1: Fig. S2 showed that the corrected slack angle increased for all of the patients who has received injection, demonstrating that the it could represent the alleviation of spasticity after BoNT-A injection.

### Diagnostic accuracy of corrected slack angle

**Fig. 7:**
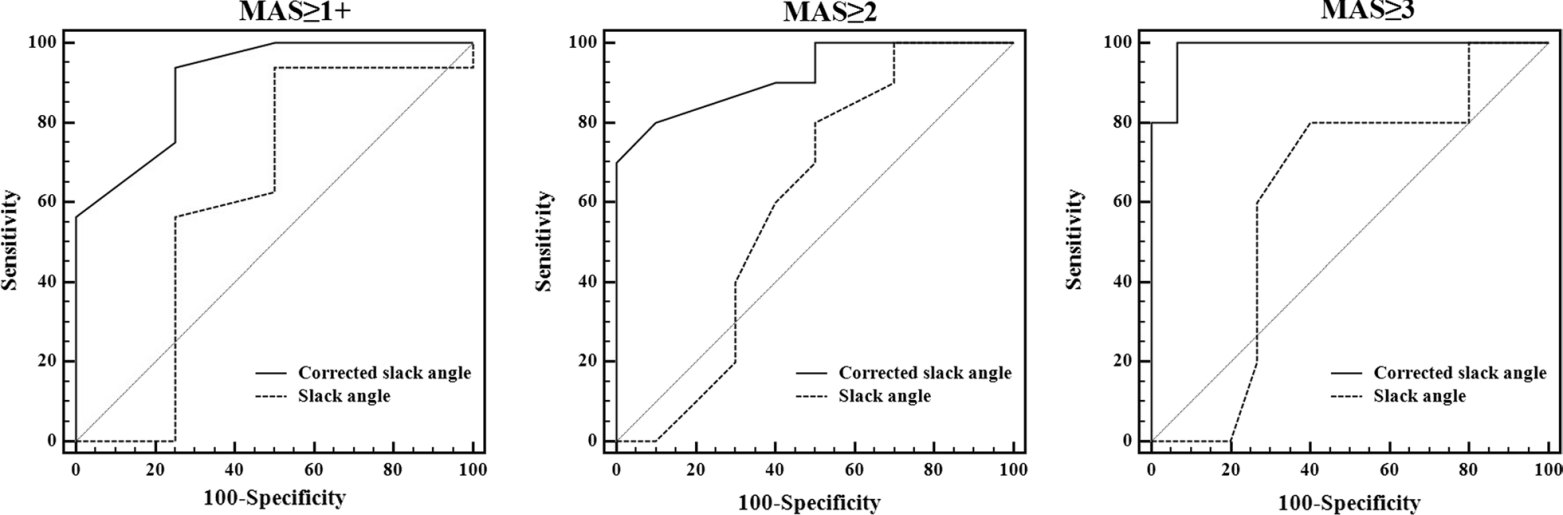
Areas under ROC curves (AUROCs) of corrected slack angle and slack angle for classifying spasticity of MAS ≥ 1+, 2 and 3 in stroke patients. The AUROCs of corrected slack angle are significantly higher than those of slack angle for classifying spasticity of MAS ≥ 2 and 3

The comparison of AUROCs revealed that corrected slack angle was significantly superior to slack angle in classifying the severity of spasticity with MAS ≥ 2 (*p* = 0.045) and MAS ≥ 3 (*p* = 0.008), while there was no significant difference in the AUROCs for classifying MAS ≥ 1+ (*p* = 0.235) (Fig. 7). Optimal cutoff values for different levels of spasticity were determined by analysis of the ROCs for corrected slack angle and slack angle. The sensitivity, specificity, PPV, NPV, LR+ and LR− of the optimal cutoff values for each spasticity classification were given in Table 4. The sensitivity and specificity for each level of spasticity were greater than 75% and the optimal cutoff value for classification of MAS ≥ 3 had the highest sensitivity (100% 95% CI 47.8–100.0%) and specificity (93.33% 95% CI 68.1–99.8%). In terms of predictive values, the PPV for classification of MAS ≥ 1+ and the NPV for classification of MAS ≥ 3 were greater than 90% (Table 4).

**Table 4:** Performance characteristics of corrected slack angle and slack angle for classifying different severity of spasticity based on optimal cutoff values

Method	MAS ≥ 1+ (95% CI)	MAS ≥ 2 (95% CI)	MAS ≥ 3 (95% CI)
Corrected slack angle			
Cutoff value	3.75	− 6.75	− 10.25
AUROC	0.906 (0.691–0.990)	0.920 (0.709–0.993)	0.987 (0.808–1.000)
Sensitivity (%)	93.75 (69.8–99.8)	80.00 (44.4–97.5)	100.00 (47.8–100.0)
Specificity (%)	75.00 (19.4–99.4)	90.00 (55.5–99.7)	93.33 (68.1–99.8)
PPV (%)	93.7 (68.6–99.9)	88.9 (48.9–99.8)	83.3 (31.1–99.8)
NPV (%)	75.0 (13.2–99.8)	81.8 (46.3–98.1)	100.0 (76.8–100.0)
LR+	3.75 (2.1–6.7)	8.00 (5.5–11.6)	15.00 (13.1–17.2)
LR−	0.083 (0.007–1.1)	0.22 (0.02–2.1)	0
Slack angle			
Cutoff value	0.25	− 3.25	− 6.75
AUC	0.617 (0.377–0.822)	0.605 (0.365–0.813)	0.620 (0.379–0.824)
Sensitivity (%)	93.75 (69.8–99.8)	80.00 (44.4–97.5)	80.00 (28.4–99.5)
Specificity (%)	50.00 (6.8–93.2)	50.00 (18.7–81.3)	60.00 (32.3–83.7)
PPV (%)	88.2 (62.6–98.7)	61.5 (31.6–86.1)	2.00 (1.1–3.7)
NPV (%)	66.7 (4.0–99.8)	71.4 (25.8–97.2)	0.33 (0.05–2.1)
LR+	1.87 (0.7–5.0)	1.60 (0.8–3.2)	40.0 (12.2–73.8)
LR−	0.13 (0.01–1.1)	0.40 (0.1–1.6)	90.0 (53.0–99.8)

Data in parentheses are 95% confidence intervals

*MAS* modified Ashworth scale, *AUROC* area under the receiver operator characteristic curve, *PPV* positive predictive value, *NPV* negative predictive value, *LR+* positive likelihood ratio, *LR−* negative likelihood ratio

## Discussion

In this study, we investigated the spasticity of MG with 2D-SWE in stroke patients. Reliability of slack angle determined with 3-SD criterion was found to be good, and the corrected slack angle was significantly correlated with MAS score. We also identified the optimal cutoff values of corrected slack angle for different levels of spasticity. The sensitivity and specificity for classification of MAS ≥ 3 were the highest, demonstrating that the corrected slack angle had advantage in distinguishing severe spasticity. In addition, the high PPV for classification of MAS ≥ 1+ and the high NPV for classification of MAS ≥ 3 indicated the clinical utility of corrected slack angle for early identification of mild spasticity and precise confirmation of severe spasticity that may need interventional therapy.

The shear modulus obtained in our experiment was comparable to those of related study. Mathevon et al. reported that the shear modulus of MG was 32.21 ± 21.1 kPa when the ankle was at maximal dorsiflexion (averaged angle of − 2 ± 8.5°) and the knee was fully extended [11]. The shear modulus we measured at a similar position (PF 5°, i.e. − 5°) was 27.12 ± 7.78 kPa. As for slack angle, the median values reported by Le Sant et al. were − 10.7° when the knee flexed at 90° and − 25.2° when the knee extended [18]. In our study, the slack angle was − 7.8 ± 9.99° when the knee flexed at 30°. Because the fluctuations at the very beginning of the curve were taken into account in the 3-SD method, the slack angle was expected to be latter (dorsiflexed) than that using visual detection. These may suggest that the slack angle could be affected by the knee position and the method that used to determine. Therefore, a standard protocol should be established in future.

In our study, the ankle was passively moved at a velocity of 1.75°/s. This slow velocity was consistent with other studies [16, 22] and used in order to obtain enough values to draw a curve of modulus-angle relationship with better resolution. We used CPM instead of dynamometer to passively move the ankle because CPM was a common equipment possessed by most of the rehabilitation facilities. It would be more suitable to be promoted in clinical scenario, while dynamometer was limited to be used owing to its high expense. Its efficiency was supported by the intra- and interobserver reliability that were as relatively good as all above 0.75 except PF 5°. We speculated that the reliability was lower at PF 25° and PF 5° because the muscle fibers were over-shortened and wrinkled when the ankle was plantarflexed. It may lead to deteriorated quality of imaging (e.g., flashes, artifacts or blank in the sample box) and unstable measurement values.

Several previous studies didn't reveal correlation between muscle elasticity and clinical spasticity assessment [14, 25, 26]. The first reason might be that shear modulus could not be simply interpreted as the severity of spasticity. Alternatively, it would be more suitable to be regarded as a parameter of estimating the stiffness of biological tissues. Therefore, we used slack angle to evaluate the spasticity. Second, the intrinsic mechanical property of skeletal muscle was individual-specific [27] and would be a confounding factor that need to be self-corrected in order to distinguish the spasticity from the intrinsic mechanical property. As anticipation, our results demonstrated that the corrected slack angle significantly correlated with MAS scores.

However, we admitted that self-correction could not completely exclude the non-neural contributors since the bilateral structural changes were not synchronous following stroke. The spastic muscles were lack of use much more than those in the unaffected side. It had also been reported that the contributions of these non-neural factors to stiffness may increase over time [28]. Hence, several precautions had been taken to offset the discrepancy of structural changes between bilateral muscles. First, to reduce the impact of immobilization, only stroke patients with hemiparesis less than 6 months were recruited. Actually most of them were less than 3 months with an average of 79.15 ± 35.37 days since stroke. Second, the enrolled patients had been receiving physical therapy to maintain their functional activity. We believed that the corrected slack angle could primarily represent the spasticity, especially in the early stage of stroke rehabilitation. Given the early stage is the time window of spasticity management, it would make sense to quantitate the spasticity using corrected slack angle.

Another method to verify the feasibility of corrected slack angle was to compare before and after BoNT-A injection. BoNT-A injection was effective in reducing spasticity by inhibiting the release of acetylcholine in neuromuscular junctions [29]. It has been confirmed that the shear modulus of MG decreased after BoNT-A injection in children with cerebral palsy [30]. And our results showed that the corrected slack angle became larger 2 weeks after BoNT-A injection as expectation. However, its statistical significance needs to be examined with further randomized controlled experiments. Further experiments may also include testing whether it is superior to calculate the dosage of BoNT-A based on spasticity quantitation with corrected slack angle. Our study had another limitations. First, the sample size of participants was limited. Second, only the thickest part of the MG was measured while other regions of MG and other muscles of triceps surae were not included because of patients’ intolerance of long-time examination. Third, constant velocity was applied to ankle movement. But increased passive resistance had been reported at higher velocity [31]. The force torque became greater as the velocity of passive ankle dorsiflexion changed from 10 to 211°/s. Therefore, the effect of velocity (i.e. whether corrected slack angle is more sensitive to represent the spasticity at higher velocity) should be addressed in further experiments.

## Conclusion

The reliability of slack angle determined using 3-SD criterion was good in assessing spasticity of stroke patients. A significant correlation was revealed between the corrected slack angle and MAS score. The highest sensitivity and specificity of the corrected slack angle were found in classifying severe spasticity. This study provided an objective parameter for quantitation of the post-stroke muscle spasticity. It may be of importance in clinical utility for early identification of mild spasticity and precise confirmation of severe spasticity which may need medical intervention.

## Supplementary Information

**Additional file 1: Table S1.** The actual passive range of motion, peak plantarflexion angle and peak dorsiflexion angle for each participants in the patient group. **Table S2.** The actual passive range of motion, peak plantarflexion angle and peak dorsiflexion angle for each participants in the control group. **Table S3.** Comparisons of slack angle and shear modulus in three different ankle positions between stroke patients and healthy controls. **Table S4**. Correlation between corrected slack angle, slack angle, shear modulus in three different ankle positions and MAS score. **Figure S1.** Correlation between MAS scores and shear modulus at the ankle positions of PF 25° (**a**), PF 5° (**b**) and DF 15° (**c**) in stroke patients. **Figure S2.** Changes of corrected slack angle of five stroke patients after BoNT-A injection. The triangle, hollow circle and solid circle indicate patients of MAS 1+, 2 and 3 assessed before injection, respectively.

## Data Availability

The datasets analysed during the current study are available from the corresponding author on reasonable request.

## Abbreviations

2D-SWE: Two-dimensional shear wave elastography
PF: Plantarflexion
DF: Dorsiflexion
MAS: Modified Ashworth scale
BoNT-A: Botulinum toxin A
CPM: Continuous passive motion
MG: Medial gastrocnemius
ROC: Receiver operating characteristic
ICC: Intraclass correlation coefficient
AUROCs: Areas under the ROC curves
