# Supplementary material for: Reliability and diagnostic accuracy of corrected slack angle derived from 2D-SWE in quantitating muscle spasticity of stroke patients
Source: J Neuroeng Rehabil. 2022 Feb 5;19:15. doi: 10.1186/s12984-022-00995-8 (PMC8817514; doi:10.1186/s12984-022-00995-8)
Supplement: Supplementary file 1 — Additional file 1: Table S1. The actual passive range of motion, peak plantarflexion angle and peak dorsiflexion angle for each participants in the patient group. Table S2. The actual passive range of motion, peak plantarflexion angle and peak dorsiflexion angle for each participants in the control group. Table S3. Comparisons of slack angle and shear modulus in three different ankle positions between stroke patients and healthy controls. Table S4. Correlation between corrected slack angle, slack angle, shear modulus in three different ankle positions and MAS score. Figure S1. Correlation between MAS scores and shear modulus at the ankle positions of PF 25° (a), PF 5° (b) and DF 15° (c) in stroke patients. Figure S2. Changes of corrected slack angle of five stroke patients after BoNT-A injection. The triangle, hollow circle and solid circle indicate patients of MAS 1+, 2 and 3 assessed before injection, respectively. [file 12984_2022_995_MOESM1_ESM.docx]

**Table S1** The actual passive range of motion, peak plantarflexion angle and peak dorsiflexion angle for each participants in the patient group

| Patients | Actual PROM(°) | Peak PF angle(°) | Peak DF angle(°) |
| --- | --- | --- | --- |
| 1 | 65 | 42 | 23 |
| 2 | 62 | 40 | 22 |
| 3 | 64 | 41 | 23 |
| 4 | 63 | 41 | 22 |
| 5 | 65 | 42 | 23 |
| 6 | 62 | 40 | 22 |
| 7 | 67 | 43 | 24 |
| 8 | 65 | 42 | 23 |
| 9 | 66 | 41 | 25 |
| 10 | 65 | 41 | 24 |
| 11 | 65 | 42 | 23 |
| 12 | 65 | 43 | 22 |
| 13 | 68 | 43 | 25 |
| 14 | 65 | 42 | 23 |
| 15 | 66 | 43 | 23 |
| 16 | 64 | 40 | 24 |
| 17 | 64 | 41 | 23 |
| 18 | 64 | 42 | 22 |
| 19 | 65 | 41 | 24 |
| 20 | 64 | 41 | 23 |
| mean ± SD | 64.70 ± 1.45 | 41.55 ± 1.00 | 23.15 ± 0.93 |

*PROM*, passive range of motion; *PF*, plantarflexion; *DF*, dorsiflexion; *SD,* standard deviation

**Table S2** The actual passive range of motion, peak plantarflexion angle and peak dorsiflexion angle for each participants in the control group

| Controls | Actual PROM(°) | Peak PF angle(°) | Peak DF angle(°) |
| --- | --- | --- | --- |
| 1 | 69 | 43 | 26 |
| 2 | 70 | 44 | 26 |
| 3 | 70 | 45 | 25 |
| 4 | 67 | 43 | 24 |
| 5 | 68 | 43 | 25 |
| 6 | 71 | 44 | 27 |
| 7 | 70 | 42 | 28 |
| 8 | 75 | 46 | 29 |
| 9 | 66 | 42 | 24 |
| 10 | 69 | 43 | 26 |
| 11 | 70 | 42 | 28 |
| 12 | 65 | 41 | 24 |
| 13 | 70 | 46 | 24 |
| 14 | 68 | 42 | 26 |
| 15 | 69 | 44 | 25 |
| 16 | 69 | 43 | 26 |
| 17 | 71 | 44 | 27 |
| 18 | 64 | 41 | 23 |
| 19 | 66 | 42 | 24 |
| 20 | 67 | 42 | 25 |
| mean ± SD | 68.70 ± 2.47 | 43.10 ± 1.45 | 25.60 ± 1.60 |

*PROM*, passive range of motion; *PF*, plantarflexion; *DF*, dorsiflexion; *SD,* standard deviation

**Table S3** Comparisons of slack angle and shear modulus in three different ankle positions between stroke patients and healthy controls

| Measurement | Controls | Patients | *p* value |
| --- | --- | --- | --- |
| Shear modulus (kPa) |  |  |  |
| PF 25° | 11.10±1.54 | 14.24±4.40 | 0.006 |
| PF 5° | 18.54±3.04 | 27.12±7.78 | ＜0.001 |
| DF 15° | 53.14±7.84 | 97.72±34.87 | ＜0.001 |

Data are means ± standard deviation. *PF*, plantarflexion; *DF*, dorsiflexion

**Table S4** Correlation between corrected slack angle, slack angle, shear modulus in three different ankle positions and MAS score

| Measurement | Correlation with MAS | |
| --- | --- | --- |
| Shear modulus (kPa) | r | *p* value |
| PF 25° | 0.010 | 0.966 |
| PF 5° | 0.326 | 0.161 |
| DF 15° | 0.297 | 0.204 |
| Slack angle | -0.219 | 0.354 |
| Corrected slack angle | -0.849 | ＜0.001 |

*MAS*, modified Ashworth scale; *PF*, plantarflexion; *DF*, dorsiflexion


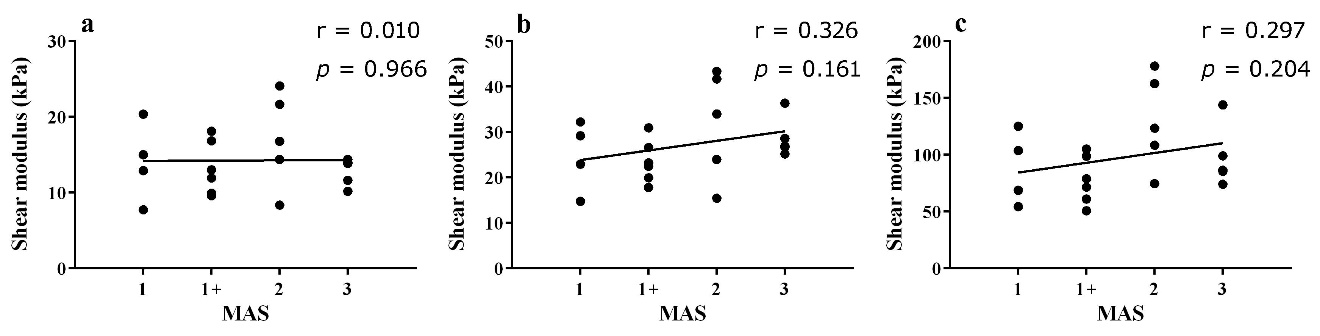


**Fig. S1** Correlation between MAS scores and shear modulus at the ankle positions of PF 25° (**a**), PF 5° (**b**) and DF 15° (**c**) in stroke patients.

**

**

**Fig. S2** Changes of corrected slack angle of five stroke patients after BoNT-A injection. The triangle, hollow circle and solid circle indicate patients of MAS 1+, 2 and 3 assessed before injection, respectively.
